# A budget impact analysis of substituting sitagliptin with liraglutide in type 2 diabetes from a private health insurance perspective in Egypt

**DOI:** 10.1186/s12962-021-00335-y

**Published:** 2022-01-15

**Authors:** Gihan Hamdy Elsisi, Ayman Afify, Ashraf Abgad, Ibtissam Zakaria, Nabil Nasif, Hani Naiem Ibrahim, Nabil Raafat, João L. Carapinha

**Affiliations:** 1 HTA Office, LLC, Cairo, Egypt; 2 Cairo University & American University in Cairo, 51 Helmy Hassan Aly St., Mostafa Elnahas, Nasr City, Cairo, Egypt; 3 Market Access and Public Affairs, Novonordisk, Cairo, Egypt; 4 Faculty of Medicine, Alex University, Alexandria, Egypt; 5 Faculty of Medicine, Cairo University, Giza, Egypt; 6 Faculty of Medicine, Ain Shams University, Cairo, Egypt; 7 Diabetes and Neuropathy Clinic, Cairo, Egypt; 8 Faculty of Medicine, Al-Azhar University, Cairo, Egypt; 9 School of Pharmacy, North-Eastern University, Boston, USA

## Abstract

**Introduction:**

Type 2 diabetes mellitus causes a sizable burden globally from both health and economic points of view. This study aimed to assess the budget impact of substituting sitagliptin with liraglutide versus other glucose-lowering drugs from the private health insurance perspective in Egypt over a 3-year time horizon.

**Methods:**

Two budget impact models were compared with the standard of care (metformin, pioglitazone, gliclazide, insulin glargine, repaglinide, and empagliflozin) administered in addition to liraglutide or sitagliptin versus the standard of care with placebo. A gradual market introduction of liraglutide or sitagliptin was assumed, and the existing market shares for the other glucose-lowering drugs were provided and validated by the Expert Panel. The event rates were extracted from the LEADER and TECOS trials. Direct and mortality costs were measured. Sensitivity analyses were performed.

**Results:**

The estimated target population of 120,574 type 2 diabetic adult patients was associated with cardio vascular risk. The budget impact per patient per month for liraglutide is EGP29 ($6.7), EGP39 ($9), and EGP49 ($11.3) in the 1st, 2nd, and 3rd years, respectively. The budget impact per patient per month for sitagliptin is EGP11 ($2.5), EGP14 ($3.2), and EGP18 ($4.1) in the 1st, 2nd, and 3rd years, respectively. Furthermore, adoption of liraglutide resulted in 203 fewer deaths and 550 avoided hospitalizations, while sitagliptin resulted in 43 increased deaths and 14 avoided hospitalizations. The treatment costs of liraglutide use are mostly offset by substantial savings due to fewer cardiovascular-related events, avoided mortality and avoided hospitalizations over 3 years.

**Conclusion:**

Adding liraglutide resulted in a modest budget impact, suggesting that the upfront drug costs were offset by budget savings due to fewer cardiovascular-related complications and deaths avoided compared to the standard of care. Sitagliptin resulted in a small budget impact but was associated with increased deaths and fewer hospitalizations avoided.

## Key points

The upfront drug costs of adding liraglutide were offset by budget savings due to fewer CV-related complications and deaths avoided compared to the standard of care (modest budget impact). Sitagliptin resulted in a small budget impact but was associated with increased deaths and fewer hospitalizations avoided. This study will help to guide reimbursement decisions in Egypt.

## Introduction

Diabetes mellitus is a chronic disease characterized by high levels of plasma glucose and deficiency in insulin production or utilization. Diabetes causes a sizable burden globally from both health and economic points of view. In 2017, the International Diabetes Federation (IDF) estimated that the number of people suffering from diabetes worldwide was 425 million, a number that is expected to increase to 629 million by 2045 [1]. Of the current diabetic population, 79% are from low- and middle-income countries, and the highest prevalence is among people aged 40 and 59 [1]. Diabetes costs public health systems around the world approximately 727 billion USD and caused 4 million deaths in 2017 [1].

The IDF ranked Egypt as the ninth highest country in the number of type 2 diabetes mellitus (T2DM) patients [2]. The number of T2DM patients has increased threefold over 20 years ago, with a current prevalence of 15.6% among the 20 to 79 age group [2]. T2DM is a metabolic disease associated with microvascular and macrovascular complications. Optimal glucose control is associated with a reduced risk of microvascular complications (retinopathy, nephropathy, and neuropathy) and benefits for macrovascular complications (reduced rates of heart attacks, strokes and improved blood flow to legs) [3].

Liraglutide is a subcutaneous injection (approved in Egypt for treating T2DM), a human glucagon-like peptide 1 (GLP-1) with an established plasma glucose-lowering effect, thus reducing the risk of microvascular complications [3]. The effects of glycemic control on macrovascular complications were evaluated in the LEADER clinical trial [3]. The primary outcome in this trial (first occurrence of death from CV causes, nonfatal myocardial infarction, or nonfatal stroke) occurred in the liraglutide group [13.0%] was fewer than in the placebo group [14.9%] (hazard ratio, 0.87; 95% confidence interval [CI] 0.78 to 0.97; P < 0.001 for noninferiority; P = 0.01 for superiority) [3]. Sitagliptin, an orally administered dipeptidyl peptidase 4 (DPP-4) inhibitor, had been registered in Egypt [4]. Sitagliptin prolongs the action of incretin hormones, by inhibiting the breakdown of GLP-1 and glucose-dependent insulinotropic polypeptide [4]. The primary composite outcome for TECOS trial, a noninferiority trial, was CV death, nonfatal stroke, nonfatal MI and hospitalization for unstable angina [4]. Sitagliptin (11.4%) was noninferior to placebo (11.6%) for the primary outcome (hazard ratio, 0.98; 95% CI 0.88 to 1.09; P < 0.001) [4].

The Egyptian health care system is fragmented and has different payers and providers. The private health insurance system is a key payer among multiple payers in Egypt. In order to provide the private health insurers with evidence to build their decisions on, and giving that efficient spending in healthcare is well known to be a direct predictor of better health outcomes and national wealth, we conducted our study to evaluate the budget impact of substituting sitagliptin with liraglutide versus other glucose-lowering drugs (metformin, pioglitazone, gliclazide, insulin glargine, repaglinide, and empagliflozin) from the private health insurance perspective in Egypt over a 3-year time horizon.

## Methodology

### Population and treatment mix

**Fig. 1:**
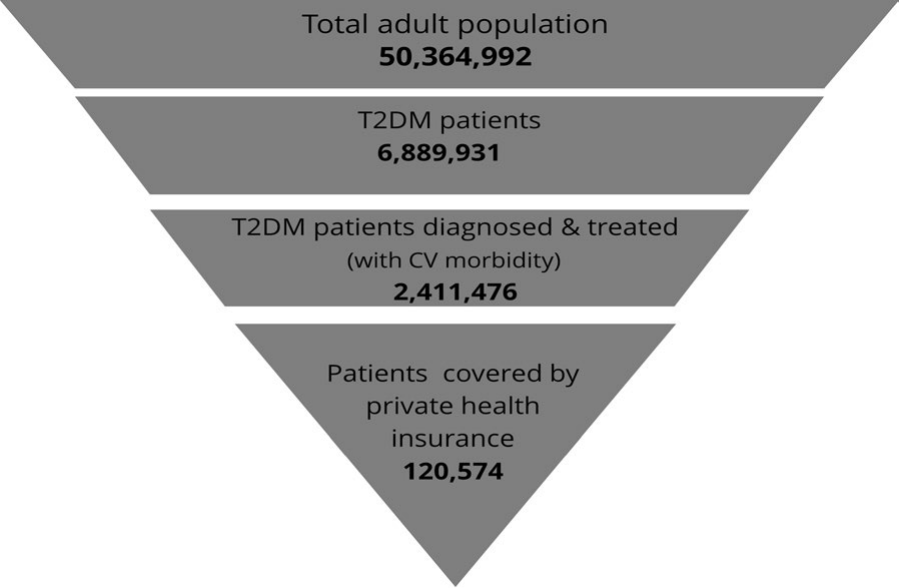
The target population. *T2DM* type 2 diabetes mellitus, *CV* cardiovascular

Two budget impact models were constructed. The first model assessed the budget impact of liraglutide plus the existing therapy (glucose-lowering drugs; metformin, pioglitazone, gliclazide, insulin glargine, repaglinide, and empagliflozin) versus the existing therapy alone, while the second model assessed the budget impact of the use of sitagliptin plus the existing therapy (mentioned above) versus the existing therapy alone. The target population of T2DM patients was estimated with the current Egyptian adult population and the prevalence of T2DM in Egypt [2, 5]. The estimated target population was then narrowed to a group of diagnosed and treated patients at risk of cardiovascular events, as demonstrated in Fig. 1 [6]. This study focused exclusively on the proportion of this patient group covered by private health insurance companies [7]. The number of targeted patients was estimated to be 120,574. A gradual market introduction of liraglutide or sitagliptin was assumed, and the existing market shares for the other glucose-lowering drugs were provided and validated by the Expert Panel.

### Clinical parameters

The event rates for cardiovascular complications were extracted from the LEADER trial [3]. This trial was a double-blind randomized trial included 9340 participants at randomization to investigate the cardiovascular safety of liraglutide versus the standard of care (metformin, thiazolidinedione, sulfonylureas, insulin, meglitinides and FGABG (SGLT2i)) in T2DM patients with a high risk for cardiovascular (CV) events [3]. The events considered in this study included mortality, myocardial infarction, stroke, heart failure (HF), coronary revascularization, and microvascular complications (retinopathy and nephropathy). The event rate difference per complication (liraglutide vs. standard of care) was multiplied by the number of patients on liraglutide to determine the number of cardiovascular events avoided. Similarly, sitagliptin inputs were extracted from the TECOS trial, a randomized, double-blind study that assigned 14,671 patients to add either sitagliptin or placebo to their existing therapy [4]. The median follow-up was 3 years to assess the cardiovascular implications of adding sitagliptin to the standard of care for T2DM patients. The clinical parameters extracted from the two studies were different but we did not standardize these parameters because we were measuring all the direct medical costs in the real-world practice. All clinical parameters were included in the model (Table 1).

**Table 1:** The model inputs parameters

Parameter	Mean value	Low value	High value	Sources
Population data				
Total adult population	50,364,992	40,291,994	60,437,990	[5]
T2DM prevalence	13.7%	10.9%	16.4%	[2]
Proportion of patients with Type 2 Diabetes diagnosed and treated (with CV morbidity)	35%	28%	42%	[6]
Proportion of patients covered by private insurance companies in Egypt	5%	4%	6%	[7]
GLP-1RA adoption year 1	3%	2.4%	3.6%	IMS data
GLP-1RA adoption year 2	4%	3.2%	4.8%	IMS data
GLP-1RA adoption year 3	5%	4%	6%	IMS data
Clinical parameters from the LEADER trial				
MI^a^	− 0.80%	− 0.00640	− 0.00960	[3]
Ischemic stroke^a^	− 0.40%	− 0.00320	− 0.00480	[3]
HF (hospitalization)^a^	− 0.60%	− 0.00480	− 0.00720	[3]
Coronary revascularization^a^	− 0.70%	− 0.00560	− 0.00840	[3]
Retinopathy^a^	0.30%	0.00240	0.00360	[3]
Nephropathy^a^	− 1.50%	− 0.01200	− 0.01800	[3]
Unstable angina pectoris (hospitalization)^a^	− 0.10%	− 0.00080	− 0.00120	[3]
All-cause mortality^a^	− 1.40%	− 0.01120	− 0.01680	[3]
Clinical parameters from the TECOS trial				
MI^b^	− 0.10%	− 0.00080	− 0.00120	[4]
Ischemic stroke^b^	− 0.20%	− 0.00160	− 0.00240	[4]
Unstable angina pectoris (hospitalization)^b^	− 0.20%	− 0.00160	− 0.00240	[4]
Severe hypoglycemia^b^	0.30%	0.00240	0.00360	[4]
CV death^b^	0.30%	0.00240	0.00360	[4]
Acute pancreatitis^b^	0.10%	0.00080	0.00120	[4]
HF (hospitalization)^b^	0.10%	0.00080	0.00120	[4]
Pancreatic cancer^b^	− 0.10%	− 0.00080	− 0.00120	[4]
Treatment costs per unit (Egyptian Pounds)				
Liraglutide	87.60 20.37 USD	70.08 16.29 USD	105.12 24.44 USD	[8]
Sitagliptin 100	11.00 2.55 USD	8.80 2.04 USD	13.20 3.06 USD	[8]
Metformin 1000	0.90 0.209 USD	0.72 0.16 USD	1.08 0.25 USD	[8]
Insulin (glargine) 100 IU	126.00 29.30 USD	100.80 23.44 USD	151.20 35.16 USD	[8]
SU (gliclazide 60 mg)	1.58 0.36 USD	1.26 0.29 USD	1.89 0.43 USD	[8]
TZD (pioglitazone 15 mg)	5.13 1.19 USD	4.11 0.95 USD	6.16 1.43 USD	[8]
Novonorm 2 mg (repaglinide)	1.47 0.34 USD	1.17 0.27 USD	1.76 0.40 USD	[8]
SGLT2i (empagliflozin)	16.53 3.84 USD	13.23 3.07 USD	19.84 4.61 USD	[8]
Event costs excluding medicines (in Egyptian Pounds)				
Non-fatal MI	76,719 17,841 USD	61,375 14,273 USD	92,062 21,409 USD	[8]
Non-fatal stroke	65,928 15,332 USD	52,742 12,265 USD	79,113 18,398 USD	[8]
HF (hospitalization)	161,249 37,499 USD	128,999 29,999 USD	193,498 44,999 USD	[8]
Coronary revascularization	67,919 15,795 USD	54,335 12,636 USD	81,502 18,953 USD	[8]
Retinopathy	20,000 4651 USD	16,000 3720 USD	24,000 5581 USD	[8]
Nephropathy	215,695 50,161 USD	172,556 40,129 USD	258,834 60,193 USD	[8]
Unstable angina pectoris (hospitalization)	76,719 17,841 USD	61,375 14,273 USD	92,062 21,409 USD	[8]
Acute pancreatitis	55,883 12,996 USD	44,706 10,396 USD	67,059 15,595 USD	[8]
Severe hypoglycemia	8059 1874 USD	6447 1499 USD	9670 2248 USD	[8]
Pancreatic cancer	92,136 21,426 USD	73,708 17,141 USD	110,563 25,712 USD	[8]
Mortality	750,000 174,418 USD	600,000 139,534 USD	900,000 209,302 USD	[8]

*T2DM* type 2 diabetes mellitus, *CV* cardiovascular, *GLP1-RA* Glucagon like peptide 1 receptor agonist, *HF* heart failure, *MI* myocardial infarction, *SU* sulphonyl urea, *TZD* thiazolidinediones, *SGLT2i* Sodium/glucose cotransporter-2 inhibitors

^a^Rate difference with and without liraglutide

^b^Rate difference with and without sitagliptin

### Costs

Direct medical costs were estimated for drug acquisition, drug administration, complication management, follow-up and adverse event costs. All unit costs of medications were extracted from the private health insurance payer lists as fixed reimbursement amounts and multiplied by the drug utilization to obtain monthly and annual costs for liraglutide, sitagliptin, metformin, thiazolidinediones, sulfonylureas, insulin, meglitinides and SGLT2i. The drug utilization proportions were extracted from the LEADER and TECOS trials [3, 4].

The average management cost for MI and unstable angina included the cost of diagnostic tests (CT scan and ECG test), either percutaneous coronary intervention (PCI) or coronary artery bypass graft (CABG) intervention, and a 7-day hospital stay including appropriate treatment. The management cost for stroke included brain magnetic resonance imaging (MRI), 6 days in the ICU and 6 days in the general ward, including appropriate treatment. The heart failure management costs were composed of diagnostic tests (echo Doppler and ECG tests), 4 days in the ICU and 10 days in general ward costs including appropriate treatment. The costs of retinopathy included corrective surgery and measures for better glycemic control, and the costs of nephropathy included measures to control blood pressure, hemodialysis to counteract kidney damage, and glomerular filtration rate (GFR) tests every 3 months.

The drug-related adverse events included were acute pancreatitis, pancreatic cancer, and severe hypoglycemia [3, 4]. Pancreatitis management costs comprised the cost of 7 days in the intensive care unit (ICU), 3 days of hospitalization in a ward, pain killers and antibiotics. For the average management cost of pancreatic cancer, it was assumed to be limited to the annual price of erlotinib, an epidermal growth factor receptor (EGFR) tyrosine kinase inhibitor approved for the management of pancreatic cancer, which is given daily via the oral route. The cost of severe hypoglycaemia management was calculated as the summation of 4 days of hospitalization, admission to the emergency room, glucose intravenous administration, and laboratory tests (creatinine test, GFR tests, liver function tests, and glucose tests). All unit costs were extracted from private insurance hospitals [8].

Mortality costs were also considered in our analysis. Even though indirect costs are not typically included in budget impact analysis, according to the ISPOR task force for good research practices for budget impact analysis, we considered mortality, as private insurance companies have to pay compensation to the patients’ family in case of death based on contracted life insurance policies [9]. To measure the cost of mortality, each patient’s life was estimated to have the stated value mentioned in the life insurance policy agreements.

The total event cost for each complication was multiplied by the event rate in each treatment arm (from the LEADER and TECOS trials) to determine the total cost per event. The medication costs were included with event costs, and the total cost difference of liraglutide vs without liraglutide was calculated as the budget impact of adopting liraglutide in T2DM patients with cardiovascular risks from the private health insurance perspective in Egypt. Similarly, the budget impact of sitagliptin vs without sitagliptin was calculated in T2DM patients with cardiovascular risks from the private health insurance perspective. Both liraglutide and sitagliptin were compared to placebo/standard of care. No direct comparison was made between the two interventions. All unit costs in this study were calculated in Egyptian Pounds (EGP) set in 2020 and were exchanged to USD using the purchasing power parity rate. The time horizon for the study was 3 years.

### Sensitivity analyses

To investigate the robustness of our study, one-way sensitivity analyses were conducted. All hazard ratios for the CV and non-CV outcomes were varied across its confidence intervals. Cost parameters were varied from 10 to 20% more or less from their original value to investigate the impact they would have on the results and to confirm which parameter has the highest impact on our conclusion.

## Results

**Fig. 2:**
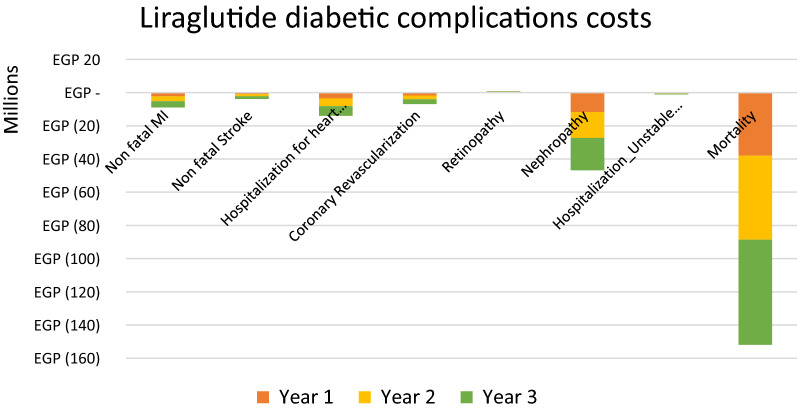
Liraglutide associated savings in diabetic complications costs over 3-year horizon. *MI* myocardial infarction

**Fig. 3:**
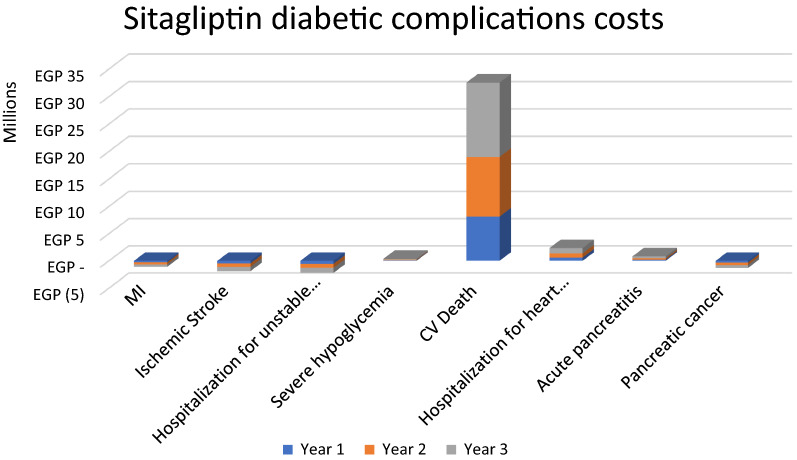
Sitagliptin associated increase in diabetic complications costs over 3-year horizon. *MI* myocardial infarction, *CV* cardiovascular

The estimated target population of 120,574 T2DM adult patients associated with CV risk in Egypt was modeled in the budget impact analysis to compare treatment with liraglutide and sitagliptin, both in addition to the standard of care. The annual results from the perspective of private health insurers over a 3-year horizon (Figs. 2 and 3) suggest that liraglutide use results in EGP232.5 million ($54 million) budget savings in medical (diabetic complications management) costs, while sitagliptin use results in a budget increase of EGP29 million ($6.7 million) in medical (diabetic complications management) costs due to an increased number of complications associated with sitagliptin. The liraglutide scenario resulted in a significant reduction in nephropathy (− 1.5% difference), mortality (− 1.4% difference), lower event rates for HF, coronary revascularization, MI, unstable angina pectoris, and stroke (− 0.6%, − 0.7%, − 0.8%, − 0.10% and − 0.4%, respectively), and a low increase in cases of retinopathy (0.3% difference). The sitagliptin scenario resulted in an increase in CV mortality, severe hypoglycaemia, HF hospitalization and acute pancreatitis (0.3%, 0.3%, 0.1%, and 0.1%, respectively) and lower event rates for unstable angina pectoris, stroke, MI and pancreatic cancer (− 0.2%, − 0.2%, − 0.1% and − 0.1%, respectively).

The budget impact per patient per month (PPPM) for liraglutide was EGP29 ($6.7), EGP39 ($9), and EGP49 ($11.3) in the 1st, 2nd, and 3rd years, respectively (Table 2). The budget impact PPPM for sitagliptin is EGP11 ($2.5), EGP14 ($3.2), and EGP18 ($4.1) in the 1st, 2nd, and 3rd years, respectively (as shown in Table 3). Furthermore, adoption of liraglutide resulted in 203 fewer deaths and 550 avoided hospitalizations, while sitagliptin resulted in 43 increased deaths and 14 avoided hospitalizations. The treatment costs of liraglutide use are mostly offset by substantial savings due to fewer CV-related events, avoided mortality and avoided hospitalizations over 3 years.

**Table 2:** Base case results of liraglutide versus without liraglutide (in Egyptian Pounds)

Parameters	Year 1	Year 2	Year 3	Cumulative
Cost of liraglutide acquisition	114,072,451 26,528,477 USD	152,096,602 35,371,302 USD	190,120,752 44,214,128 USD	456,289,805 106,113,908 USD
Cost of standard of care by substitution^a^	(13,000,266) 3,023,317 USD	(17,333,688) 4,031,090 USD	(21,667,110) 5,038,862 USD	(52,001,064) 12,093,270 USD
Drug cost	101,072,185 23,505,159 USD	134,762,914 31,340,212 USD	168,453,642 39,175,265 USD	404,288,741 94,020,637 USD
Non-fatal myocardial infarction	(2,220,074) 516,296 USD	(2,960,099) 688,395 USD	(3,700,124) 860,493 USD	(8,880,297) 2,065,185 USD
Non-fatal stroke	(953,906) 221,838 USD	(1,271,875) 295,784 USD	(1,589,844) 369,731 USD	(3,815,625) 887,354 USD
Hospitalization for heart failure	(3,499,637) 813,869 USD	(4,666,183) 1,085,158 USD	(5,832,729) 1,356,448 USD	(13,998,549) 3,255,476 USD
Coronary revascularization	(1,719,736) 399,938 USD	(2,292,982) 533,251 USD	(2,866,227) 666,564 USD	(6,878,946) 1,599,754 USD
Retinopathy	217,033 50,472 USD	289,377 67,296 USD	361,721 84,121 USD	868,131 201,890 USD
Nephropathy	(11,703,224) 2,721,680 USD	(15,604,298) 3,628,906 USD	(19,505,373) 4,536,133 USD	(46,812,895) 10,886,719
Hospitalization_unstable angina pectoris	(277,509) 64,536 USD	(370,012) 86,049 USD	(462,515) 107,561 USD	(1,110,037) 258,148 USD
Mortality	(37,980,744) 8,832,731 USD	(50,640,992) 11,776,974 USD	(63,301,240) 14,721,218 USD	(151,922,975) 35,330,924 USD
Medical costs	(58,137,798) 13,520,418 USD	(77,517,064) 18,027,224 USD	(96,896,330) 22,534,030 USD	(232,551,192) 54,081,672 USD
Total costs PPPM	29 6.74 USD	39 9.06 USD	49 11.39 USD	39 9.06 USD

*PPPM* per patient per month

^a^We substituted the market share of the glucose lowering drugs with liraglutide

**Table 3:** Base case results of sitagliptin versus without sitagliptin (in Egyptian Pounds)

Parameter	Year 1	Year 2	Year 3	Cumulative
Cost of sitagliptin acquisition	14,324,166 3,331,201 USD	19,098,888 4,441,601 USD	23,873,610 5,552,002 USD	57,296,665 13,324,805 USD
Cost of standard of care by substitution^a^	(5,823,714) 1,354,352 USD	(7,764,952) 1,805,802 USD	(9,706,190) 2,257,253 USD	(23,294,856) 5,417,408 USD
Drug cost	8,500,452 1,976,849 USD	11,333,936 2,635,799 USD	14,167,420 3,294,748 USD	34,001,809 7,907,397 USD
MI	(277,509) 64,536 USD	(370,012) 86,049 USD	(462,515) 107,561 USD	(1,110,037) 258,148 USD
Ischemic stroke	(476,953) 110,919 USD	(635,938) 151,413 USD	(794,922) 184,865 USD	(1,907,813) 443,677 USD
Hospitalization for unstable angina	(555,019) 129,074 USD	(740,025) 172,098 USD	(925,031) 215,123 USD	(2,220,074) 516,296 USD
Severe hypoglycemia	87,453 20,337 USD	116,605 27,117 USD	145,756 33,896 USD	349,814 81,352 USD
CV death	8,138,731 1,892,728 USD	10,851,641 2,523,637 USD	13,564,551 3,154,546 USD	32,554,923 7,570,912 USD
Hospitalization for heart failure or CV death	583,273 135,644 USD	777,697 180,859 USD	972,121 226,074 USD	2,333,091 542,579 USD
Acute pancreatitis	202,139 47,009 USD	269,519 62,678 USD	336,898 78,348 USD	808,556 188,036 USD
Pancreatic cancer	(333,276) 77,506 USD	(444,367) 103,341 USD	(555,459) 129,176 USD	(1,333,102) 310,023 USD
Medical costs	7,368,839 1,713,683 USD	9,825,119 2,284,911 USD	12,281,399 2,856,139 USD	29,475,358 6,854,734 USD
Total costs PPPM	11 2.55 USD	14 3.25 USD	18 4.18 USD	14 3.25 USD

*PPPM* per patient per month

^a^We substituted the market share of the glucose lowering drugs with sitagliptin

Liraglutide results in total initial savings of EGP58 million ($13 million), EGP77 million ($17 million), and EGP96 million ($22 million) in the 1st, 2nd and 3rd years in medical costs, respectively, due to avoided complications and hospitalizations. The total cumulative savings over the 3 years from a private health insurance perspective are estimated at EGP232.5 million ($54 million) (Table 2). Sitagliptin results in a total increased medical cost of EGP7 million ($1.6 million), EGP9 million ($2 million), and EGP12 million ($2.7 million) in the 1st, 2nd and 3rd years, respectively, due to increased complications and hospitalizations. The total cumulative medical costs over the 3 years from a private health insurance perspective are estimated at EGP29 million ($6.7 million) (Table 3).

### Sensitivity analyses

**Fig. 4:**
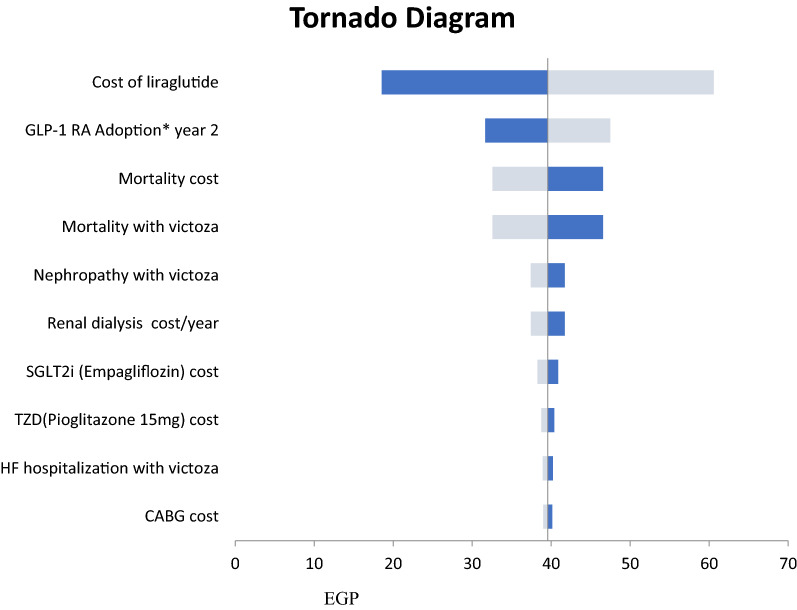
One-way sensitivity analysis results for liraglutide. *GLP1-RA* Glucagon like peptide 1 receptor agonist, *SGLT2i* Sodium/glucose cotransporter-2 inhibitors, *TZD* thiazolidinediones, *HF* heart failure, *CABG* Coronary artery bypass graft. The light grey bar corresponds with the upper range, and the dark blue bar with the lower range of an input

**Fig. 5:**
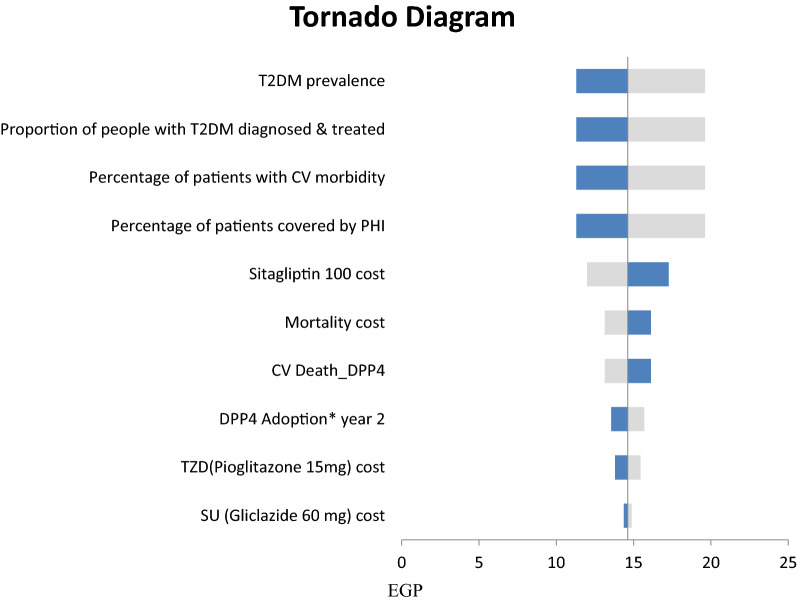
One-way sensitivity analysis results for sitagliptin. *T2DM* type 2 diabetes mellitus, *CV* cardiovascular, *PHI* private health insurance, *DPP4* dipeptidyl peptidase 4, *TZD* thiazolidinediones, *SU* sulphonyl urea. The light grey bar corresponds with the upper range, and the dark blue bar with the lower range of an input

The results of one-way deterministic sensitivity analyses (Fig. 4) suggest that the drug acquisition costs of liraglutide and its market share had the largest impact on the liraglutide model results. T2DM prevalence and the target patients diagnosed and treated with T2DM had the largest impact on the sitagliptin model results (Fig. 5).

We conducted another scenario analysis (without mortality cost) in the liraglutide budget impact model. We found that it had the same conclusion, but it resulted in budget savings of EGP80 million ($18 million) instead of EGP232 million ($54 million) if we included the mortality cost.

## Discussion

Liraglutide was approved by the European Medicines Agency in 2009 and United States Food and Drug Administration in 2010, and is sold in more than 80 countries to treat T2DM patients [10, 11]. Our results demonstrate that the upfront costs of liraglutide 1.8 mg are mostly offset by budget savings due to fewer CV-related events and premature mortality avoided (550 avoided hospitalizations and 203 avoided deaths, respectively).

Prior analyses on liraglutide use among T2DM patients also reported cost savings. In a budget impact study conducted from the Algerian healthcare payer’s perspective, liraglutide 1.2 mg resulted in cost savings compared to insulin glargine among patients insufficiently controlled on oral antidiabetics [12]. Authors reported that more patients reached the target HbA1c level without the need for intensified treatment regimens, unlike basal insulin, which in 79% of cases requires intensification to a basal-bolus regime or twice daily premix therapy, both of which have higher direct costs [12]. A study in Italy used real-world market consumption data and found that adding liraglutide versus standard of care increased the cost per patient between €8.04 and €25.00. This study did not consider the cost of complications and thus did not offset the elevated drug acquisition cost with the cost savings from the reduced rates of complications associated with liraglutide [13]. Another study assessed the budget impact and the cost-effectiveness of liraglutide versus the standard of care from the US healthcare payer perspective. Over the lifetime of T2DM patients included in the analysis and with confirmed cardiovascular disease or high cardiovascular risk, liraglutide use was budget neutral and cost-effective [14].

In the United Kingdom, a study on the cost-effectiveness of liraglutide (1.2 and 1.8 mg, daily) versus dapagliflozin (10 mg, daily) among T2DM patients concluded that both doses of liraglutide may be cost-effective treatments as a second or third addition to standard of care for patients who are not eligible for SGLT-2i therapy [15]. In Italy, a cost-effectiveness study of liraglutide 1.8 mg versus lixisenatide 20 μg (both are GLP-1 receptor agonists) for treating T2DM patients unable to reach acceptable blood glucose levels on metformin concluded that liraglutide 1.8 mg is likely to be cost-effective versus lixisenatide 20 μg in Italian settings [16]. In France, a study comparing liraglutide, sitagliptin and glimepiride as add-ons for patients not reaching the target HbA1c level found that while all fell below the willingness-to-pay threshold, liraglutide was the most cost-effective [17]. The findings of this study were of great significance, as they included CV death and all-cause death outcomes, which is the case with our model. Last, in Spain, a study comparing 1.8 mg liraglutide and sitagliptin as intensifications for patients on metformin above the target HbA1c levels concluded that 1.8 mg liraglutide is cost-effective compared to sitagliptin in Spanish settings [18].

When considering the ISPOR Special Task Force in defining the elements of value in health care that were not captured in our model due to lack of local data [19], we found that the addition of liraglutide not only provided high-quality adjusted life years (QALYs), life years gained (LYsG) and productivity values as innovative treatment of Egyptian T2DM patients when compared to standard of care but also provided the following novel health values: value of hope, real option value, adding more value in severity of disease and as a scientific spillover.

Our study was modeled on the best available evidence from the LEADER and TECOS trials. The budget impact model simulated a patient cohort covered by private health insurance in Egypt and integrated local clinical practice and epidemiological inputs validated by an expert panel. We also included various sensitivity analyses to ensure the robustness of the model and to detect any uncertainties. Our study was limited by the use of an international clinical trial with results that may not be specific to Egypt. Variations exist in treatment patterns between countries [3]. Second limitation, these results could not be generalized to the Egyptian public health care system as the unit costs are varied between the public and the private health care system. Third limitation, we did not conduct probabilistic sensitivity analyses because they are not mandatory according to Egyptian pharmacoeconomic guidelines [20]. Furthermore, ISPOR Task Force on Good Research Practices—Budget Impact Analysis mentioned in their report that sensitivity analysis should be in the form of alternative scenarios chosen from the perspective of the decision-maker and their usefulness depends on the amount and quality of available data and the needs of the decision-maker [9]. Fourth limitation, we did not conduct indirect treatment comparisons i.e. network meta-analysis for a matter of simplification and better understanding by policy makers and private health insurers. Our results would be strengthened with clinical parameters specific to Egypt. However, such variations may be negligible because the standard of care of Egypt does not differ compared to countries included in the clinical trials, and Egyptian clinical practice is based on international treatment guidelines (American Diabetes Association Guidelines) [21]. Another strength of our study was the inclusion of mortality costs, as our analysis was conducted from a private health insurance perspective, and around 85% of the private insurance companies pay life insurance to the families of dead patients.

## Conclusion

The adoption of liraglutide resulted in 203 deaths avoided and 550 hospitalizations avoided. Adding liraglutide resulted in a modest budget impact of EGP 29 ($6.7)–EGP 49 ($11.3) PPPM, suggesting that the upfront drug costs were offset by budget savings due to fewer CV-related complications and deaths avoided. Sitagliptin resulted in a budget impact of EGP 11($2.5)–EGP 18 ($4.1) PPPM but was associated with 43 deaths and 14 hospitalizations avoided compared to the standard of care in Egypt from the private health insurance perspective.

## Data Availability

This article is a budget impact analysis, and thus there are no underlying data used for this research apart from the data extracted from the articles included in this review. Cost data used to inform the budget impact analysis are available from the corresponding author upon reasonable request.
